# Prevalence, Infection Intensity and Molecular Diagnosis of Mixed Infections with *Metastrongylus* spp. (Metastrongylidae) in Wild Boars in Uzbekistan

**DOI:** 10.3390/pathogens11111316

**Published:** 2022-11-09

**Authors:** Abdurakhim E. Kuchboev, Jürgen Krücken

**Affiliations:** 1 Institute of Zoology of Uzbekistan Academy of Sciences, Bogishamol Str. 232B, Tashkent 100053, Uzbekistan; 2 Institute for Parasitology and Tropical Veterinary Medicine, Freie Universität Berlin, Robert-von-Ostertag-Str. 7, 14163 Berlin, Germany

**Keywords:** *Metastrongylus*, lungworms, wild boars, earthworms

## Abstract

The aim of the present study was to characterize the diversity of *Metastrongylus* spp. in wild boars and the earthworm intermediate host species contributing to the maintenance of the life cycle. Here, wild boars were subjected to parasitological necropsies, and lungworm species were identified morphologically, followed by confirmation using ITS-2 sequencing and a phylogenetic analysis. Earthworms were collected from wild boar habitats and investigated for the presence of larvae. The prevalence of *Metastrongylus* spp. in wild boars was 78.8%, and many individuals were positive for all three detected species, *Metastrongylus pudendotectus*, *Metastrongylus salmi* and *Metastrongylus elongatus*. The phylogenetic analysis did not clearly resolve all species, except for *M. pudendotectus*. Age group and season had no influence on prevalence, while intensity was significantly higher in autumn than in spring and summer (Kruskal–Wallis followed by Dunn’s test). Three out of six investigated earthworm species were positive for metastrongyloid larvae (prevalence of 10.4–16.7%), but neither their phylogenetic relationship nor ecological microhabitats were able to explain these differences. Further sequence data should be used to improve the resolution in phylogenetic trees to determine potential cryptic species in the genus, while the application of deep sequencing approaches might provide insights into species-specific epidemiology and pathology.

## 1. Introduction

The superfamily of Metastrongyloidea (Lane, 1917) is composed of approximately 200 nematode species divided into eight families. Several species infecting wild and domestic animals have a worldwide distribution [1,2,3,4,5,6]. Among these metastrongyloid nematodes, the family Metastrongylidae contains only a single genus, *Metastrongylus* Molin, 1861 [7], which comprises all lungworms parasitizing wild and domestic pigs, where these lungworms reside in the bronchi and bronchioles. Metastrongylids are widely distributed in feral pig populations throughout the world and are frequently found in mixed infections under natural conditions [7,8,9,10,11,12,13].

Wild boars live in small groups, constantly migrating from one place to another. They are omnivorous, feeding on vegetative underground (roots, rhizomes, tubers and bulbs) and the above-ground parts of plants, as well as various fruits, berries and seeds. Animals play a significant role in the diet of the wild boar, which is dominated by earthworms, mollusks, the larvae and adults of soil insects, small reptiles, amphibians and even small rodents, and they also scavenge on carrion and dead fish. Usually, wild boars obtain food by digging up the top layers of soil [14].

To date, seven species of the genus *Metastrongylus* have been described: *Metastrongylus elongatus* Dujardin, 1845 (syn. *Metastrongylus apri* Gmelin, 1790); *Metastrongylus salmi* Gedoelst, 1923; *Metastrongylus pudendotectus* Vostokov, 1905; *Metastrongylus confusus* Jansen, 1964; *Metastrongylus asymetricus* Noda, 1973; *Metastrongylus madagascariensis* Chabaud and Gretillat, 1956; and *Metastrongylus tschiauricus* Kojawa, 1956 [4]. Kotlan [15] indicated that *M. tschiauricus* from wild boars in Georgia is closely related to *M. pudendotectus* and possibly identical to it.

Gasso et al. [16] developed a morphological identification key for the five most common *Metastrongylus* species in order to avoid further misclassifications of *Metastrongylus* species. The identification of these worms is based on the morphology of the female’s posterior body and the length of the male’s spicules [4]. The species validity of these parasites has been clearly demonstrated, but it nevertheless remains difficult to identify species only by their morphological characteristics.

Metastrongylosis is an invasive disease of wild boars and domestic pigs caused by *Metastrongylus* spp. in the bronchi and bronchioles of the lungs. However, several cases of the infection of humans with *M. elongatus* and *M. salmi* have been described, indicating that these species are at least zoonotic [17,18,19]. 

Helminthological studies of wild boars in Central Asia, including Kazakhstan and Tajikistan, were carried out at the end of the 19th century and in the last century [14,20,21]. Three species of nematodes of the genus *Metastrongylus* (*M. elongatus*, *M. salmi* and *M. pudendotectus*) were reported in Kazakhstan [14]. In the Republic of Tajikistan, where there are relatively many wild boars and a high intensity of infection, the same three species of metastrongylids were recorded [21]. The available data on the species of the genus *Metastrongylus* occurring in Uzbekistan are fragmentary, which is reflected in the small number of publications [22,23,24]. In Uzbekistan, three species of these nematodes, namely, *M. elongatus*, *M. salmi* and *M. pudendotectus*, were found, but the dominant species was *M. pudendotectus* [25].

*Metastrongylus* spp. females shed fully larvated eggs that are transported up the trachea before they are swallowed and passed with feces [26]. First-stage larvae hatch and continue development in earthworms (Annelida, Clitellata, Opistopora and Lumbricidae) as obligate intermediate hosts [27]. The final hosts acquire the parasites by ingesting infected earthworms, which are very abundant in their environment [1]. Various species of earthworms can serve as intermediate hosts for *Metastrongylus* spp., and they allow for the completion of the parasite’s life cycle. Data regarding earthworms as intermediate hosts of *Metastrongylus* spp. are very limited [28,29,30,31,32,33,34,35]. In particular, the role of different earthworm species in the circulation of metastrongylids in Uzbekistan has not been studied.

In recent years, the first and second internal transcribing spacers (ITS-1 and ITS-2) of ribosomal DNA have been proven to be useful genetic markers for pulmonary and intestinal Strongylida [36,37,38,39,40,41]. Molecular approaches, such as ITS-2 sequencing, single-strand conformation polymorphism analyses and the random amplification of polymorphic DNA (RAPD), have been used for the identification of *Metastrongylus* spp. found in the lungs of wild boars (*Sus scrofa* L., 1758) to evaluate genetic similarities and to perform phylogenetic analyses [37,42,43]. 

The current study initially focused on the identification of ITS-2, which has future diagnostic potential to identify the sympatric assemblages of boar hosts and metastrongylid parasites from Uzbekistan. The goal of our research was to study the infection of definitive and intermediate hosts and to use molecular diagnostics to unequivocally identify the *Metastrongylus* species endemic in Uzbekistan.

## 2. Materials and Methods

### 2.1. Specimens and Morphological Comparisons

#### 2.1.1. Collection of Adult Lungworms from Wild Boars

Between 2012 and 2020, the Institute of Zoology of the Academy of Sciences of the Republic of Uzbekistan subjected the lungs of 33 wild boars (shot and dead) to helminthological autopsy. The lungs were delivered by hunting farms from the Bekabad district and the western spurs of the Chatkal ridge (Akhangaran district) of the Tashkent region, the Bukhara and Karakul hunting farms of the Bukhara region and the Arnasay hunting farm of the Jizzakh region of Uzbekistan. Adult worms were recovered at necropsy from boars as specified by Kontrimavichus et al. [4]. Adult worms and voucher specimens (heads and tails from individual adult worms for definitive identification) were preserved in 70% ethanol.

The *Metastrongylus* species were differentiated based on morphological features under an ML 2000 series microscope (Meiji Techno, Saitama, Japan). Adult nematodes were identified based on the morphology of the female’s posterior body, a pair of massive trilobed lips and long filiform spicules and on the male’s atypical bursa. The female has a relatively long vagina. The females and males of the different species were identified based on small differences in the morphology of the vulva and the spicules, respectively [1,4,16].

#### 2.1.2. Collection of Third-Stage Larval Lungworms from Earthworms

More than 500 specimens of earthworms were studied regarding their potential role as intermediate hosts of metastrongylid nematodes. The collection of earthworms was carried out in juniper forests near springs, tugays, reed thickets and grasslands, which are all well-known places for rookeries and the feeding of wild boars in the Tashkent, Jizzakh and Bukhara regions of Uzbekistan [44]. For a quantitative analysis of the earthworms in places and on paths visited by the wild boars, the method of the manual sorting of soil samples was used, including plots with an area of 25 cm × 25 cm [44,45]. Initially, the litter layer was examined at the designated site, and then layer-by-layer excavations were carried out as follows: from the surface to a depth of 10 cm, from 10 to 20 cm and from 20 to 30 cm. Adult and juvenile earthworms were selected from all soil layers. The collected lumbricides, with labels indicating the sample number, date, place of sampling and layer depth, were placed in containers with soil. Under laboratory conditions, the soil was washed off the worms, and, taking into account morphological features, they were divided into two groups: the first was fixed in a 2% formalin solution to determine the species, and the second was used to isolate metastrongylid larvae.

The species identification of earthworms (Lumbricidae) was established in accordance with the guide developed by Perel [46]. Furthermore, the species identification of the collected Lumbricidae was confirmed at the Department of Zoology of the Karshi State University. The infection of earthworms with the larvae of metastrongylids was determined using generally accepted methods [47]. For this, to detect, identify and count the nematode larvae, compressors and stereomicroscopes (MBS-10) were used. The collected earthworms were pre-killed with a 1% formalin solution. Then, the cuticle was cut with scissors, and the esophagus, goiter and muscular stomach with the blood vessels surrounding them were separated. These organs were examined using the compressor method under a microscope for the presence of metastrongylus larvae. In addition, earthworms were investigated using the method of digestion in artificial gastric juice. The number of earthworms found to be infected by larvae was counted. The size of the invasive larvae of *Metastrongylus* is 0.570 mm × 0.03 mm, and the posterior end ends in a tip (younger larvae at the caudal end of the button); not far from the caudal end, there is a small cuticular spine; the anterior end is blunt and cut off.

### 2.2. DNA Extraction, PCR Amplification and Sequencing

DNA was extracted using a DNA Purification kit (Qiagen, New Dehli, India) and eluted twice with 100 µL of the AE buffer provided in the kit. PCR amplification used 0.25 µM of each of the primers NC1 (ACGTCTGGTTCAGGGTTGTT) and NC2 (TTAGTTTCTTTTCCTCCGCT) [36], 0.2 U Phusion DNA Polymerase (Thermo Scientific, Waltham, MA, USA), 0.4 mM dNTP mix (Thermo Scientific) and 2 µL template DNA in 20 µL HF buffer. After denaturation at 98 °C for 30 s, 40 cycles of 98 °C for 10 s, 55 °C for 30 s and 72 °C for 30 s were used, followed by a final incubation at 72 °C for 10 min. PCR products were purified with DNA Clean & ConcentratorTM–5 columns (Zymo Research), and Sanger sequencing from both ends was performed by Syntol JSC (Moscow, Russia). 

### 2.3. Phylogenetic Analysis

The sequences of both strands were compared and edited using BioEdit [48]. Similar sequences in GenBank were identified using BLASTn searches [49]. All sequences of the genus *Metastrongylus* and two sequences of *Angiostrongylus vasorum*, one *Aleurostrongylus abstrusus* sequence and one *Protostrongylus hobmaieri* sequence, the latter to be used as an outgroup, were downloaded. The sequences were aligned using MAFFT 6.5 [50] on an online server [51] using the Q-INS-I option to consider RNA structure information for alignment. It was decided to use the “Leave gappy regions” option in order to avoid the artificial alignment of the non-homologous parts of the ITS-2 sequences. A maximum-likelihood phylogenetic tree was calculated on the IQ-TREE server using version 1.6.12 [52,53]. Modelfinder [54] was used to identify the optimal nucleic acid substitution model, including FreeRate heterogeneity models with four rate categories based on the lowest Bayesian information criterion. Node support was calculated using ultrafast bootstrapping [55], the Shimodaira–Hasegawa (SH)-like approximate likelihood ratio test [56] and an approximate Bayes test [57]. The trees were visualized using FigTree.v.1.4.4 (Andrew Rambout, Edinburgh, UK).

### 2.4. Statistical Analysis

Statistical analyses were either conducted in GraphPad Prism 5.02 or in R 4.1.1. Prevalence with 95% confidence intervals and significant differences in prevalence were calculated with the functions binom.wilson and tab2by2.test from the epitools package 0.5-10.1. Kruskal–Wallis tests, followed by Dunn’s post hoc tests and Mann–Whitney U tests, were conducted in GraphPad. If p values were corrected for multiple testing, the Bonferroni method was used.

## 3. Results

### 3.1. Prevalence, Intensity and Species Composition

As a result of the parasitological examination of the wild boars, sexually mature nematodes of three species from the genus *Metastrongylus* were found: *M. elongatus*, *M. pudendotectus* and *M. salmi*. Quantitative data on the prevalence and intensity of infection (number of worms in infected animals) for the three regions of Uzbekistan are shown in Table 1. Only seven of the pigs had no lungworms at all. The majority, 20 out of 33 pigs (60.6%), were infected by all three lungworm species. Five pigs were positive for *M. pudendotectus* and either *M. salmi* (n = 3) or *M. elongatus* (n = 2). One pig was only positive for *M. pudendotectus*, which was also the species with the highest prevalence, but the prevalence of the different species was neither significant for the complete dataset nor for the individual regions. Moreover, the differences between the regions were not significant.

**Figure 1:**
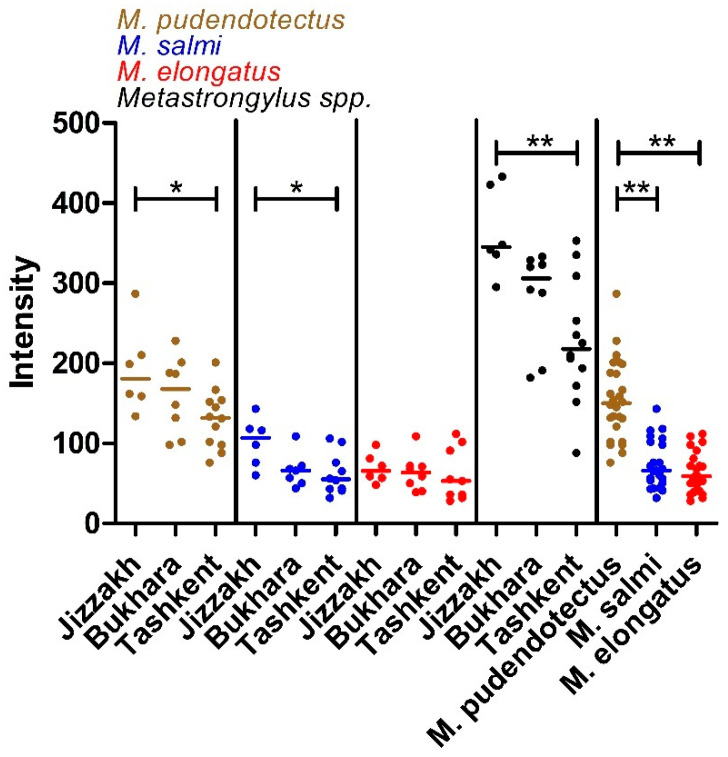
Intensity of infection in terms of number of worms of each *Metastrongylus* species or all species together. Horizontal lines indicate the median of each dataset. Datasets were compared using a Kruskal–Wallis test followed by Dunn’s post hoc test to compare the different regions or species. ** *p* < 0.01; * *p* < 0.05.

The intensity was significantly higher in Jizzakh than in Tashkent (Figure 1) for *M. podendotectus* and *M. salmi*, as well as for all *Metastrongylus* spp. together. Moreover, worm counts were significantly higher in *M. podendotectus* than in *M. salmi* and *M. elongatus*.

### 3.2. Molecular Characterization of Metastrongylus spp. from Uzbekistan

**Figure 2:**
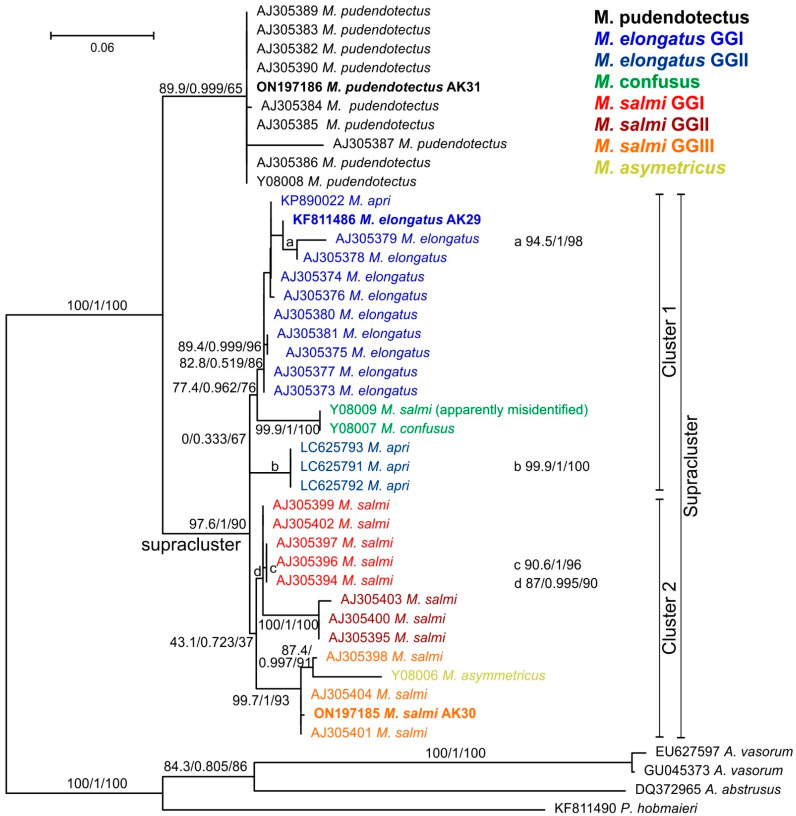
Maximum-likelihood phylogenetic tree based on ITS-2 sequences of *Metastrongylus* (*M.*) spp. Sequences of specimens in the present study are shown in bold. The tree was rooted using sequences of *Angiostrongylus vasorum*, *Aleurostrongylus abstrusum* and *Protostrongylus hobmaieri* as an outgroup. Species names are provided as given in the GenBank entries, including *M. apri*, which is a well-accepted synonym for *M. elongatus*. Branch support is indicated as SH-aLRT support (%)/aBayes support/ultrafast bootstrap support (%). The scale bar represents 0.06 substitutions per site. GG, genotype group.

In order to confirm the morphological species identification, the ITS-2 regions were successfully amplified and sequenced from adult lung nematode specimen samples, resulting in sequences of approximately 495 bp for all three species. One ITS-2 sequence for each species was deposited in GenBank (Table 2). The obtained nucleotide sequences were analyzed together with the available GenBank data by constructing a maximum-likelihood phylogenetic tree (Figure 2).

The obtained nucleotide sequences were analyzed together with the available GenBank data, resulting in the phylogram shown in Figure 2. In this tree, *M. pudendotectus* formed a very homogenous, moderately well-supported cluster clearly separated from all other species. The other species *M. elongatus*, *M. salmi*, *M. confuses* and *M. asymetricus* formed a highly supported supracluster (indicated in Figure 2), but the differences among the species within this supracluster were relatively small. There was one very poorly supported cluster 1 (0% SH-LRT support) that contained three subclusters with much higher support. These are the two clusters of *M. elongatus* (syn. *M. apri*) named in Figure 2 as the *M. elongatus* genotype group (GG) I and II. Between the two *M. elongatus* GGs, two sequences were located that only differed by a two-base-pair IN/DEL, i.e., Y08009 and Y08007. While the former is labeled as *M. salmi* in GenBank, the latter is *M. confuses*. The *M. salmi* sequence must have been obtained from a morphological misidentified specimen since it is the only *M. salmi* labeled sequence within this cluster. Thus, these sequences most likely represent *M. confuses*. A second, poorly supported large cluster 2 (43.1% SH-LRT support) was dominated by sequences labeled as *M. salmi*. This cluster 2 contained three well to highly supported subclusters with different *M. salmi* genotypes (*M. salmi* GGI-III in Figure 2). Within the *M. salmi* GGIII cluster, a single *M. asymetricus* sequence is located, connecting with a relatively long branch (Figure 2).

### 3.3. Seasonality of Infection

**Figure 3:**
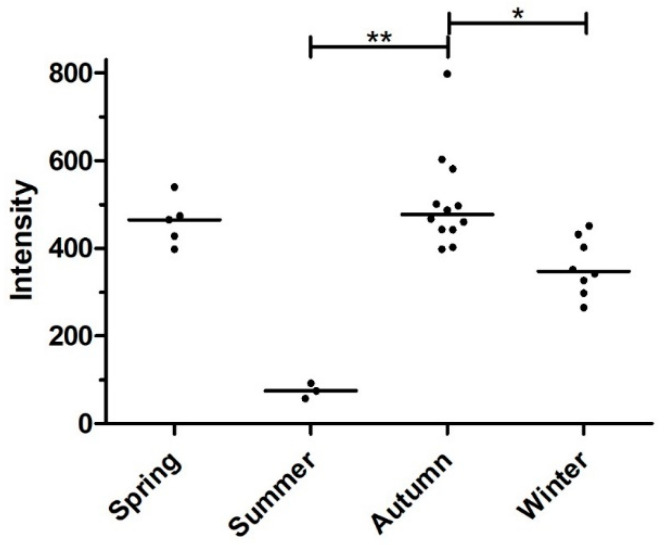
Intensity of infection measured as number of worms of *Metastrongylus* spp. in different seasons. Horizontal lines indicate the medians of each dataset. Datasets were compared using a Kruskal–Wallis test followed by Dunn’s post hoc test to compare the different seasons. ** *p* < 0.01; * *p* < 0.05.

The prevalence of infection of animals in different seasons of the year ranged from 50 to 92.3%, with the highest prevalence occurring in autumn and the lowest prevalence occurring in summer, but the differences were not significant due to the small number of animals in some of the seasons, particularly in summer (Table 3). The median intensity of infection was between 75 and 477 worms, again with the highest intensity in autumn and the lowest in summer (Figure 3). Intensity was significantly higher in autumn than in summer and winter, while spring was similar to autumn, although the differences were not significant for any of the other seasons (Figure 3).

### 3.4. Age Dynamics of Infections with Metastrongylids

**Figure 4:**
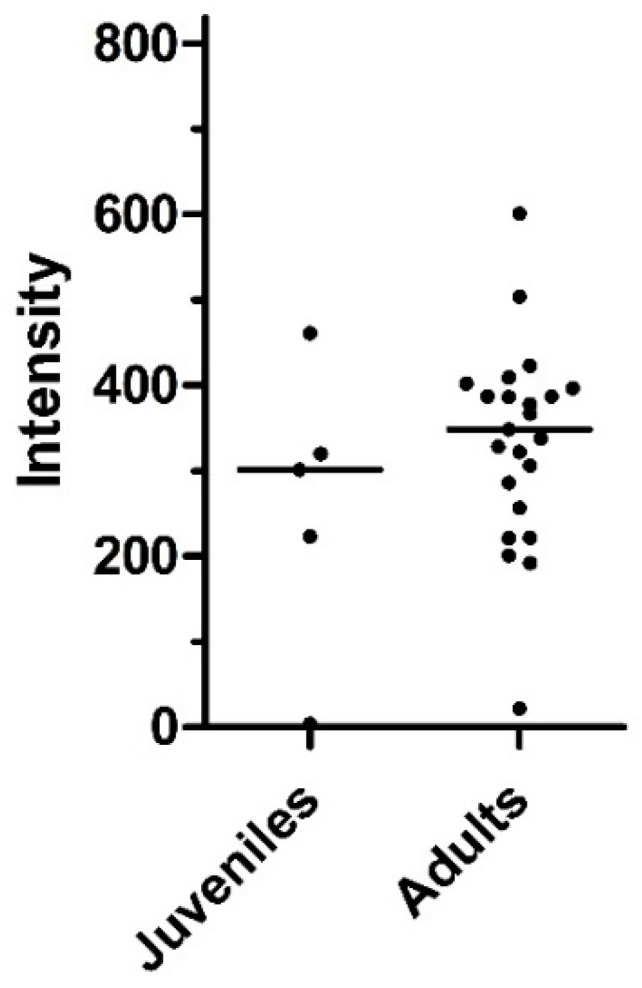
Intensity of infection with *Metastrongylus* spp. in juvenile and adult wild boars. Horizontal lines indicate the medians of each dataset. Datasets were compared using a Man–Whitney U test, but no significant differences were found.

The prevalence and intensity of the infections of the animals were further compared between juvenile and adult wild boars. In the six juveniles included in the study, the prevalence was slightly lower than in the 27 adults, but the difference was not significant (Table 4). Infection intensity was also slightly lower in juveniles than in adults (medians of 301 and 397, respectively) (Figure 4), but, again, this difference was not significant.

### 3.5. Prevalence of Metastrongylid larvae in Different Oligochaete Species

In total, six different species of earthworms were identified, and the sample size of each species was considerably high (n = 46–96). Metastrongylid larvae were found in three species, *Aporrectodea caliginosa trapezoides*, *Eisenia veneta* and *Octolasium lacteum*, all with a prevalence between 10 and 15%, while the species *Aporrectodea jassyensis*, *Eisenia veneta* and *Dendrobaena byblica* were consistently negative (Table 5). Almost all comparisons between the prevalence of positive and negative species were significant with only one exception, *A. jasiniensis* vs. *A. jassyensis*. Positive species were found to have an endogeic ecological niche, i.e., species burrowing extensive horizontal tunnel systems and feeding predominantly on soil, or to be at least partially endogeic (*Eisenia fetida*, epiendogeic). However, species negative for metastrongylid larvae were also sometimes endogeic (*A. jassyensis*) or epigeic, i.e., species dwelling on the ground and in the litter layer and feeding predominantly from plant litter [58].

## 4. Discussion

The genus *Metastrongylus* contains several parasites of pigs that are rarely investigated. Due to the obligate intermediate host, these parasites are not relevant in industrial pig production [59], and their clinical relevance has also been questioned [60]. Since wild boars are the most frequently infected hosts, these parasites have mostly been neglected by researchers in the recent past.

*Metastrongylus* spp. have been reported worldwide with variable frequencies [10,16,61,62,63,64,65,66,67,68]. In a previous study conducted by Kuchboev et al. [25], a similarly high prevalence of 84.6–92.2% was observed for the same three species of *Metastrongylus*. In this study, *M. pudendotectus* was slightly more prevalent than the other two species. Another study from Central Asia involved only 10 wild boras from two different regions of Kazakhstan. Again, the same three species were found. However, the prevalence was considerably lower, with 42.8% for *M. pudendotectus* and *M. elongatus*, but the authors did not report the prevalence of *M. salmi* [14]. In Eastern Europe, a prevalence of 100% was found in the Ryazan region of Russia, but the authors only found *M. pudendotestus* and *M. elongatus* [69]. In a national park close to Moscow, two out of five wild boars were positive for the three species *M. elongatus*, *M. pudendotectus* and *M. salmi* [70]. In Belarus, 98.4% of adult and 100% of juvenile wild boars were positive for *Metastrongylus* spp., with *M. pudendotectus* being the most prevalent species, followed by *M. elongatus* and *M. salmi* [71]. The same three species were also detected in Bulgaria [72]. In contrast, all five Metastrongylus species were found in Spain [16], Poland [73] and Switzerland [74]. In Switzerland, the overall prevalence was 77.4%, with *M. pudendotectus* being found most frequently, followed by *M. salmi*, *M. confusus* and *M. apri*, with *M. asymetricus* being the least frequently found [74]. In Poland, the order was *M. pudendotectus*, *M. salmi*, *M. asymmetricus*, *M. elongatus* and *M. confusus*. When looking into geographical regions other than Eurasia, a high prevalence of 84.5% for *Metastrongylus* spp. was described for wild boars in Morocco. Again, *M. pudendotectus* was the most prevalent species (84.5%), but with a prevalence of 72.7%, *M. confusus* was the second most frequently found species, followed by *M. salmi* (51.5%), whereas *M. elongatus* was not found. In Japan, wild boars showed 100% prevalence, and 64.3% were infected with all four species that were detected. The order of prevalence was *M. asymmetricus*, *M. salmi*, *M. pudendotectus* and *M. elongatus*. In feral pigs in Florida, *M. apri*, *M. salmi* and *M. pudendotectus* were collected during necropsies, with a prevalence of 94%, 76% and 65%, respectively [9]. Apparently, in Eastern European and Central Asian populations of wild boars, the presence of *M. pudendotectus*, *M. elongatus* and *M. salmi* (in the order of their typical prevalence) is widely observed, and the results from Uzbekistan are in line with many previous data from this region. 

In Uzbekistan, the majority of wild boars were infected with multiple *Metastrongylus* species. This was also the case for all other studies cited above regarding the prevalence and species composition of pig lungworm. The high number of *Metastrongylus* species in combination with the very high overall prevalence that was described in all the studies make it clear that co-infections must be the rule rather than the exception. However, none of the studies, including the present one, has attempted to statistically analyze whether co-infections occur more frequently than one would expect according to the prevalence of each individual *Metastrongylus* species. The simple reason for this is that the number of animals included in these studies ranged from 5 to less than 50, and, thus, this is far too low for meaningful analyses of such dependencies.

Earthworms are a major part of the feed of wild boars, and, thus, *Metastrongylus* spp. turned out to be highly prevalent (63.6–92.3%), with infections reaching very high intensities in the present study. While the prevalence of the nematodes was not significantly influenced by age group or the season of the year, there was a significant effect of the season on intensity, with autumn showing a higher intensity than summer and winter. However, these results should be considered with caution since the very low intensities in summer were due to only three infected wild boars from this season. The difference between summer and autumn was based on much higher animal numbers, but it was also only moderate. In contrast to the results shown here, the infestation of earthworms in spring, summer and autumn has been described to be approximately the same in Belarus [30]. This suggests that differences in infection burden in pigs over the year might be due to differences in the role of earthworms in their diet.

Earthworms of the family Lumbricidae that serve as intermediate hosts can have a very high prevalence of almost 100%, with an intensity of tens to a few thousand larvae in one worm as described for species such as *Aporrectodea caliginosa, Dendrobaena octaedra, Eisenia fetida* and *Lumbricus terrestis* [28]. The infection intensity of wild boars with *Metastrongylus* spp. has been shown to be the major factor influencing infections in earthworms [75]. Earthworms that become infected by metastrongylid larvae remain infected lifelong [15], which can lead to the accumulation of larvae to high infection burdens. Most frequently and intensively infected were earthworm species that live in the upper layer of soil, plant debris and humus on the surface of the earth, i.e., epigeic species. The deeper the habitat of a worm species in soil, the lower the prevalence of metastrongyloids in these worms [28]. However, the results of the present study are contradictory to this view since all entirely epigeic species with their habitat in the upper leaf litter were negative. All positive species were either endogeic or epi to epiendogeic. However, the ecological category alone was also not sufficient to explain the pattern of infection observed in the present study in earthworms since the 46 specimens of the endogeic species *A. jassyensis* were all negative.

The interaction of *Metastrongylus* spp. with wild boars and relevant lumbricid earthworm species has rarely been investigated. The earthworms collected in the present study came from typical wild boar habitats. The fact that very closely related earthworm species, such as the two *Aporectodea* and the two *Eisenia* species, differed so strongly in the prevalence of metastrongylid larvae suggests that the members of these species pairs differ in their ecological macro- or micro-habitats. Since both are considered to be endogeic, different macrohabitats with different wild boar densities or activities might be important variables. The collection of different earthworm species with sampling sites that are georeferenced on a very small scale and with reference to the surrounding vegetation and wild boar activity might help to provide explanations for the unusual distribution pattern of metastrongyloid larvae observed here. However, such investigations are very labor-intensive since the sample site should be considered the statistical unit in many of the methods needed to analyze such data.

Based on both morphologic and molecular data, all isolated lungworms from the wild boars in Uzbekistan were *M. elongatus*, *M. pudendotectus* and *M. salmi*. As discussed above, many of the wild boars were infected with multiple *Metastrongylus* species simultaneously. This makes it difficult to determine whether there are differences in the epidemiology (e.g., preference of intermediate host species) or pathogenicity of the different species. Molecular tools have the potential to improve the available data on individual *Metastrongylus* spp., e.g., tools based on non-invasive fecal samples, in the future.

The literature data on the comparative study of the ITS-2 of other representatives of lungworms show that there are small but stable species differences that allow for the information about the structure of this site to be used as a rather effective tool for resolving the controversial issues of the taxonomy of nematodes of this group [6,76]. For metastrongylids, Conole et al. [37] used sequencing and a single-strand conformation polymorphism analysis (SSCP) of the ITS-2 to identify the species *M. elongatus*, *M. pudendotectus* and *M. salmi* in wild boars, which allowed for the direct display of sequence variation within and among individuals representing each species.

In the phylogenetic tree, *M. pudendotectus* is clearly separated from the other species. The separation of the four species *M. elongatus*, *M. salmi*, *M. confusus* and *M. asymetricus* was not completely clear. Neither the sequences of *M. elongatus* nor those of *M. salmi* formed monophyletic clusters in the phylogram since *M. confusus* is located in the *M. elongatus* and *M. asymetricus* in the *M. salmi* cluster. This could be interpreted as if *M. confuses* is only a special morphotype of *M. elongatus* and *M. asymetricus* a morphotype of *M. salmi* and that these names should be considered synonyms. However, statistical support for those clusters containing all *M. elongatus* (and *M. confusus*) and all *M. salmi* (and *M. asymetricus*) was also only poor. Alternative interpretations of the tree could be that all sequences in the supracluster belong to a single, genetically quite variable species, or that each of the genotype groups represents a different species. This would mean that *M. elongatus* would be split into two species, and *M. salmi* would be split into three species. Additional sequence data are required to determine which of these alternatives is the preferable hypothesis and to also better understand the phylogenetic history of *M. asymetricus* in the genus. In previous studies, it was shown that combined analyses of nuclear (e.g., ITS-2, ITS-1 and β-tubulin isotype 1) and mitochondrial (e.g., cytochrome oxidase 1, 12S and 16S subunit mitochondrial rRNAs) genes can help to obtain both a reliable phylogenetic tree and enough resolution to discriminate between closely related, sometimes cryptic, species [41,77,78,79]. However, such analyses in the future will rely on the availability of material for rarer species, such as *M. confusus* and *M. asymetricus*.

PCR-based approaches have become an important tool for investigating parasite communities and populations but also host–parasite interactions, and they will probably also help in livestock management in the future [80]. The ability to monitor individual hosts rapidly over time makes it possible to investigate (i) the contribution of different helminth species to total parasite burden, (ii) the ecological relationships between helminth species and the magnitude and direction of genetic correlations between resistances to different nematode species in host populations and (iii) the occurrence of anthelmintic resistance in certain parasite populations. While this is already quite advanced today for the gastrointestinal parasitic nematodes of livestock, including massively parallel sequencing of amplicons, such as ITS-2 or the isotype 1 β-tubulin, to describe the parasite community or the resistance status of multiple species [81], it has not yet been described for pulmonary parasites. Such methods would allow non-invasive investigations of lungworm communities in wild boars but can also be extended to other species, including protected wildlife.

## Figures and Tables

**Table 1:** Prevalence of metastrongylids in wild boars in Uzbekistan.

		*Metastrongylus* spp.		*Metastrongylus pudendotectus*		*Metastrongylus salmi*		*Metastrongylus elongatus*	
Region	N	n	% Prevalence (95% CI)	n	% Prevalence (95% CI)	n	% Prevalence (95% CI)	n	% Prevalence (95% CI)
Jizzakh	9	6	66.7 (35.4–87.8)	6	66.7 (35.4–87.8)	6	66.7 (35.4–87.8)	6	66.7 (35.4–87.8)
Bukhara	11	8	72.7 (43.4–90.3)	8	72.7 (43.4–90.3)	7	63.6 (35.4–84.8)	8	72.7 (43.4–90.3)
Tashkent	13	12	92.3 (66.7–98.6)	12	92.3 (66.7–98.6)	11	84.6 (57.8–95.7)	11	84.6 (57.8–95.7)
Total	33	26	78.8 (62.2–89.3)	26	78.8 (62.2–89.3)	24	72.7 (55.8–84.9)	25	75.7 (59.0–87.2)

N, total number of animals; n, number of positive animals; 95% CI, 95% confidence interval. There were no significant differences in prevalence between seasons using the mid-p exact test.

**Table 2:** Origin of specimens with sequence information.

Species	Specimen ID	GenBank Accession No.	Stage	Host and Locality
*Metastrongylus elongatus*	29	KF811486	adult	*Sus scrofa*, Tashkent, Uzbekistan
*Metastrongylus salmi*	30	ON197185	adult	*Sus scrofa*, Tashkent, Uzbekistan
*Metastrongylus pudendotectus*	31	ON197186	adult	*Sus scrofa*, Tashkent, Uzbekistan

**Table 3:** Seasonal dynamics of wild boar infection with metastrongyls.

Season	N	n	% Prevalence (95% CI)
Spring	5	4	80 (37.6–96.4)
Summer	6	3	50 (18.8–81.2)
Autumn	13	12	92.3 (66.7–98.6)
Winter	9	8	88.8 (56.5–98.0)

N, total number of animals; n, number of positive animals; 95% CI, 95% confidence interval. There were no significant differences in prevalence between seasons using the mid-p exact test.

**Table 4:** Prevalence of metastrongylid infection in different age groups of wild boars.

Age Group	N	n	% Prevalence (95% CI)
Juveniles	6	5	83.3 (43.6–97.0)
Adults	27	21	77.8 (59.2–89.4)

N, total number of animals; n, number of positive animals; 95% CI, 95% confidence interval. There were no significant differences in prevalence between seasons using the mid-p exact test.

**Table 5:** Prevalence of metastrongylid infections in different species of Oligochaeta.

Oligochaeta Species	N	n	% Prevalence (95% CI)	Significant Differences	Ecology ^§^
*Aporrectodea caliginosa trapezoides*	96	16	16.7 (10.5–25.4)	a,d,e,g,j,l	Endogeic
*Aporrectodea jassyensis*	46	0	0 (0–7.7)	b,c,e,h,j,k	Endogeic
*Octolasium lacteum*	48	5	10.4 (4.5–22.2)	a,d,e,g,j,l	Endogeic
*Eisenia fetida*	64	8	12.5 (6.5–22.8)	a,d,e,g,j,l	Epigeic, epiendogeic
*Eisenia veneta*	96	0	0 (0–3.8)	b,c,f,h,i,k	Epigeic
*Dendrobaena byblica*	88	0	0 (0–4.2)	b,c,f,h,i,k	Epigeic

N, total number of animals; n, number of positive animals; 95% CI, 95% confidence interval. The following pairs indicate significant differences in the mid-p exact test: a/b comparison of *Aporrectodea caliginosa trapezoides* to other species ((b) if significant (*p* < 0.05 in mid-p exact test after Bonferroni correction) and (a) if non-significant); c/d comparison to *Aporrectodea jassyensis* (c) and other species (c/d, depending on significance); e/f comparison to Octolasium lacteum (e) and all other species (e/f, depending on significance); g/h comparison to *Eisenia fetida* (g) and all other species (depending on significance); *Octolasium lacteum*; i/j comparison to *Eisenia veneta* (i) and all other species (i/j depending on significance); l/k comparison to *Dendrobaena byblica* (l) and all other species (l/k depending on significance). ^§^ Ecological status according to http://taxo.drilobase.org/, accessed on 13 September 2022.

## Data Availability

Data are contained within the article. Sequence data were deposited in GenBank.

## References

[1] Anderson R.C. (2000). Nematode Parasites of Vertebrates: Their Development and Transmission.

[2] Boev S.N. (1975). Protostrongylids, Fundamentals of Nematology.

[3] Durette-Desset M.C., Beveridge I., Spratt D.M. (1994). The origins and evolutionary expansion of the Strongylida (Nematoda). Int. J. Parasitol..

[4] Kontrimavicius V.L., Delyamure S.L., Boev S.N. (1976). Fundamentals of Nematodology. Metastrongyloids of Domestic and Wild Animals. M..

[5] Tokobaev M.M. (1976). Helminths of wild mammals of Central Asia.

[6] Kuchboev A.E., Krücken J., Ruziev B.H., Von Samson-Himmelstjerna G. (2015). Molecular phylogeny and diagnosis of species of the family Protostrongylidae from caprine hosts in Uzbekistan. Parasitol. Res..

[7] Beveridge I., Spratt D.M., Durette-Desset M.C., Schmidt-Rhaesa A. (2014). Order Strongylida (Railliet & Henry, 1913) Handbook of Zoology.

[8] Khrustalev A.V. (1981). On the species composition of the genus *Metastrongylus*-parasites of the lungs of pigs and wild boars in the USSR. Parasitology.

[9] Forrester D.J., Porter J.H., Belden R.C., Frankenberger W.B. (1982). Lungworms of feral swine in Florida. J. Am. Vet. Med. Assoc..

[10] Morita T., Haruta K., Shibata-Haruta A., Kanda E., Imai S., Ike K. (2007). Lung worms of wild boars in the western region of Tokyo, Japan. J. Vet. Med. Sci..

[11] Da Silva D., Müller G. (2013). Parasites of the respiratory tract of *Sus scrofa scrofa* (wild boar) from commercial breeder in southern Brazil and its relationship with *Ascaris suum*. Parasitol. Res..

[12] Nagy G., Varga G., Csivincsik A., Sugar L. (2013). Occurrence of *Metastrongylus asymmetricus* (Noda, 1973) in Hungary. Magy. Allatorv. Lapja.

[13] Ruziev B.K., Kuchboev A.E., Karimova R.R. (2020). Ecology of nematodes of the genus *Metastrongylus* Molin, 1861-parasites of the wild boar of Uzbekistan. Bull. Karshi State Univ..

[14] Baitursunov K.K. (2010). To the study of the ecology of wild boar helminths (*Sus scrofa* Linnaeus, 1758) in Kazakhstan. Proceedings of the National Academy of Sciences of the Republic of Kazakhstan.

[15] Kotlan A. (1960). Die Helminthosen der Haus- und Nutztiere unter Berücksichtigung der Helminthosen des Menschen.

[16] Gassó D.L., Rossi G., Mentaberre E., Casas R., Velarde P., Nosal E., Serrano J., Segales P., Fernandez-Llario P., Feliu C. (2014). An identification key for the five most common species of *Metastrongylus*. Parasitol. Res..

[17] Miloshev B. (1956). Case of triple infection with *Metastrongylus elongatus*, *Thaeniarhynchus saginatus* and *Enterobius vermicularis*. Suvrem. Meditsina.

[18] Beaver P.C., Jung R.C., Cupp E.W. (1984). Clinical Parasitology.

[19] Calvopina M., Caballero H., Morita T., Korenaga M. (2016). Human pulmonary infection by the zoonotic *Metastrongylus salmi* nematode. The first reported case in the Americas. Am. J. Trop. Med. Hyg..

[20] Shol V.A. (1964). Helminthiases of pigs in Kazakhstan.

[21] Melnikova T.G. (1961). Some information about the helminth fauna of the wild boar *Sus scroga nigripes* in Tajikistan. Collection Natural foci of diseases and questions of parasitology. Alma-Ata.

[22] Koshchanov E.K. (1972). Helminthes of wild mammals of Uzbekistan. Ph.D. Thesis.

[23] Shapolatov Z.S. (1979). Pig Parasites in Uzbekistan (Helminths).

[24] Karimova R.R., Kuchboev A.E., Umarov D.K. Pulmonary nematodes of wild mammals of Uzbekistan. Proceedings of the Actual Problems of Studying and Preserving the Animal World of Uzbekistan.

[25] Kuchboev A.E., Umarov D.K., Karimova R.R., Ruziev B.K. Metastrongilids of wild boars in Uzbekistan. Proceedings of the European Multicolloquium of Parasitology: Program&Abstract Book EMOP XI.

[26] Alicata J.E. (1934). Life history of *Metastrongylus salmi* and remarks on the eggs of the swine lungworm. Proc. Helminthol. Soc. Wash..

[27] Hobmaier A., Hobmaier M. (1929). Die Entwicklung der Larve des Lungenwurmes *Metastrongylus elongatus* (*Strongylus paradoxus*) des Schweines und ihr Invasionsweg, sowie vorläufige Mitteilung über die Entwicklung von *Choerostrongylus brevivaginatus*. Münch. Tierärtzl. Wschr..

[28] Breza M.K. (1961). Epizootologickemu vyznamu horizontsnej migraete dazdoviek (najna *E. foetida*) pri metastrongylosach osipanych. Folia Vet..

[29] Rose J. (1959). *Metastrongylus apri* the pig lungworm: Observations on the free-living embryonated egg and the larva in the intermediate host. Parasitology.

[30] Bobkova A.F. (1966). The study of the epizootology of pig metastrongilosis in Belarus and the search for new methods of therapy—Achievements of veterinary science-in production. Trans. Belarusian Sci. Vet..

[31] Kolevatova A.I. (1972). Infection of lumbricides metastrongylus. Mater. Vses. Obs. Helminthol..

[32] Tiunov V.I. (1966). Some questions of the relationship between metastrongylus and their intermediate hosts. Teach materials. Trans. Vses. Inst. Helminthol..

[33] Ustinov I.D. (1963). Infection of various species of earthworms with metastrongylid larvae in farms that is unfavorable for pig metastrongilosis. Trans. Vses. Inst. Helminthol..

[34] Burenkov S.N., Krotenkov V.P. (2013). Ecological and epizootological features of Lumbricidae-intermediate hosts of wild boar metastrongylids. Russ. Parasitol. J..

[35] Antipov A.A., Bakhur T.I., Feshchenko D.V., Romanishina T.A., Avramenko N.V., Goncharenko V.P., Zghozinska O.A., Solovyova L.M., Koziy N.V., Pidborska R.V. (2018). Earthworms (Lumbricidae) as Intermidiate Hosts of Lung Nematodes (Metastrongylidae) of Swine in Kyiv and Zhytomyr Regions of Ukraine. Vestn. Zool..

[36] Gasser R.B., Chilton N.B., Hoste H., Beveridge I. (1993). Rapid sequencing of rDNA from single worms and eggs of parasitic helminths. Nucleic Acids Res..

[37] Conole J.C., Chilton N.B., Jarvis T., Gasser R.B. (2001). Mutation scanning analysis of microsatellite variability in the second internal transcribed spacer (precursor ribosomal RNA) for three species of *Metastrongylus* (Strongylida: Metastrongyloidea). Parasitology.

[38] Santín-Durán M., de la Fuente C., Alunda J.M., Rosenthal B.M., Hoberg E.P. (2002). Identical ITS-1 and ITS-2 sequences suggest *Spiculopteragia asymmetrica* and *Spiculopteragia quadrispiculata* (Nematoda: Trichostrongylidae) constitute morphologically distinct variants of a single species. J. Parasitol..

[39] Chilton N.B., Huby-Chilton F., Gasser R.B., Beveridge I. (2006). The evolutionary origins of nematodes within the order Strongylida are related to predilection sites within hosts. Mol. Phylogenetics Evol..

[40] Kutz S.J., Asmundsson I., Hoberg E.P., Appleyard G.D., Jenkins E.J., Beckmen K., Branigan M., Butler L., Chilton N.B., Cooley D. (2007). Serendipitous discovery of a novel protostrongylid (Nematoda: Metastrongyloidea) in caribou (*Rangifer tarandus*), muskoxen (*Ovibos moschatus*) and moose (*Alces alces*) from high latitudes of North America based on DNA sequence comparisons. Can. J. Zool..

[41] Kuchboev A., Sobirova K., Karimova R., Amirov O., Samson-Himmelstjerna G., Krücken J. (2020). Molecular analysis of polymorphic species of the genus *Marshallagia* (Nematoda: Ostertagiinae). Parasites Vectors.

[42] Leignel V., Humbert J.F., Elard L. (1997). Study by ribosomal DNA ITS 2 sequencing and RAPD analysis on the systematics of four Metastrongylus species (Nematoda: Metastrongyloidea). J. Parasitol..

[43] Carreno R.A., Nadler S.A. (2003). Phylogenetic analysis of the Metastrongyloidea (Nematoda: Strongylida) inferred from ribosomal RNA gene sequences. J. Parasitol..

[44] Malevich I.I. (1950). Collection and Study of Earthworms-Soil-Formers.

[45] Gilyarov M.S. (1987). Accounting for large invertebrates (mesofauna). Quantitative Methods in Soil Zoology.

[46] Perel T.S. (1979). Distribution and Patterns of Distribution of Earthworms in the Fauna of the USSR.

[47] Kotelnikov G.A. (1974). Diagnosis of Helminthiases in Animals.

[48] Hall T.A. (1999). BioEdit: A User-Friendly Biological Sequence Alignment Editor and Analysis Program for Windows 95/98/NT. Nucleic Acids Symp. Ser..

[49] Altschul S.F., Gish W., Miller W., Myers E.W., Lipman D.J. (1990). Basic local alignment search tool. J. Mol. Biol..

[50] Katoh K., Misawa K., Kuma K., Miyata T. (2002). MAFFT: A novel method for rapid multiple sequence alignment based on fast Fourier transform. Nucleic Acids Res..

[51] Katoh K., Rozewicki J., Yamada K.D. (2019). MAFFT online service: Multiple sequence alignment, interactive sequence choice and visualization. Briefimgs Bioinform..

[52] Nguyen L.T., Schmidt H.A., Haeseler A., Minh B.Q. (2015). IQ-TREE: A Fast and Effective Stochastic Algorithm for Estimating Maximum-Likelihood Phylogenies. Mol. Biol. Evol..

[53] Trifinopoulos J., Nguyen L.T., von Haeseler A., Minh B.Q. (2016). W-IQ-TREE: A fast online phylogenetic tool for maximum likelihood analysis. Nucleic Acids Res..

[54] Kalyaanamoorthy S., Minh B., Wong T., Haeseler A., Jermiin L.S. (2017). ModelFinder: Fast model selection for accurate phylogenetic estimates. Nat. Methods.

[55] Hoang D.T., Chernomor O., Haeseler A., Minh B.Q., Vinh L.S. (2018). UFBoot2: Improving the Ultrafast Bootstrap Approximation. Mol. Biol. Evol..

[56] Guindon S., Dufayard J.-F., Lefort V., Anisimova M., Hordijk W., Gascuel O. (2010). New Algorithms and Methods to Estimate Maximum-Likelihood Phylogenies: Assessing the Performance of PhyML 3.0. Syst. Biol..

[57] Anisimova M., Gil M., Dufayard J.-F., Dessimoz C., Gascuel O. (2011). Survey of Branch Support Methods Demonstrates Accuracy, Power, and Robustness of Fast Likelihood-based Approximation Schemes. Syst. Biol..

[58] Regina M.M., Marycruz Á.J., Alix D., Frédérique R., Manuel B., José A.G., Carlos R.C., Roger G., Luc V., Isabelle B. (2019). Earthworms Building Up Soil Microbiota, a Review. Front. Environ. Sci. Eng..

[59] Thamsborg S.M., Roepstorff A., Larsen M. (1999). Integrated and biological control of parasites in organic and conventional production systems. Vet. Parasitol..

[60] Wallgren P., Pettersson E. (2022). Lungworms (*Metastrongylus* spp.) demonstrated in domestic pigs with respiratory disease: Was there a clinical relevance?. Porc. Health Manag..

[61] Fernandez-de-Mera I.G., Gortazar C., Vicente J., Hofle U., Fierro Y. (2003). Wild boar helminths: Risks in animal translocations. Vet. Parasitol..

[62] Jarvis T., Kapel C., Moks E., Talvik H., Magi E. (2007). Helminths of wild boar in the isolated population close to the northern border of its habitat area. Vet. Parasitol..

[63] Förster M., Klimpel M., Sievert K. (2009). The house fly (*Musca domestica*) as a potential vector of metazoan parasites caught in a pig-pen in Germany. Vet. Parasitol..

[64] Senlik B., Cirak V., Girisgin O., Akyol C. (2011). Helminth infections of wild boars (*Sus scrofa*) in the Bursa province of Turkey. J. Helminthol..

[65] Mansouri M., Sarkari B., Mowlavi G.R. (2016). Helminth parasites of wild boars. Iran. J. Parasitol..

[66] Cleveland C.A., DeNicola A., Dubey J.P., Hill D.E., Berghaus R.D., Yabsley M.J. (2017). Survey for selected pathogens in wild pigs (*Sus scrofa*) from Guam, Marianna Islands, USA. Vet. Microbiol..

[67] Li K., Luo H., Zhang H., Lan Y., Han Z., Shahzad M., Wang X., Qiu G., Huang S., Jiang W. (2016). First report of *Metastrongylus pudendotectus* by the genetic characterization of mitochondria genome of cox1 in pigs from Tibet, China. Vet. Parasitol..

[68] Panayotova-Pencheva M., Dakova V. (2018). Studies on the gastrointestinal and lung parasite fauna of wild boars (*Sus scrofa scrofa* L.) from Bulgaria. Ann. Parasitol..

[69] Andreyanov O.N. (2013). Helminth fauna of wild boar in the Ryazan region. Russ. Parasitol. J..

[70] Samoilovskaya N.A. (2011). Parasite fauna of wild boars in the “Losiny Ostrov” National Park (Moscow). Russ. Parasitol. J..

[71] Kaplich V.M., Yakubovsky M.V., Tereshkina N.V. (2013). About the helminthes fauna of wild boar (*Sus scrofa*) in the subzone of oak-dark coniferous forests of Belarus. Proceedings of Belarusian State Technological University.

[72] Dakova V., Panayotova-Pencheva M. (2017). Morphometric Features of Three Lungworms in Materials from Wild Boars from Bulgaria. Acta Morphol. Anthropol..

[73] Nosal P., Kowal J., Nowosad B. (2010). Structure of Metastrongylidae in wild boars from southern Poland. Helminthologia.

[74] Spieler N., Schnyder M. (2021). Lungworms (*Metastrongylus* spp.) and intestinal parasitic stages of two separated Swiss wild boar populations north and south of the Alps: Similar parasite spectrum with regional idiosyncrasies. Int. J. Parasitol. Parasites Wildl..

[75] Nagy G., Csivincsik A., Sugár L. (2014). Wild boar density drives *Metastrongylus* infection in earthworm. Acta Parasitol..

[76] Hoglund J., Wilhelmsson E., Christensson D., Morner T., Waller P.G., Mattsson J. (1999). ITS2 sequences of *Dictyocaulus* species from cattle, roe deer and moose in Sweden: Molecular evidence for a new species. Int. J. Parasitol..

[77] von Samson-Himmelstjerna G., Janssen I.J.I., Ramünke S., Goday C., Borges F.A., Koudela B., Niedźwiedź A., Tomczuk K., Studzińska M.B., Kornas S. (2021). Very low intraspecific sequence variation in selected nuclear and mitochondrial Parascaris univalens genes. Infect. Genet. Evol..

[78] Louro M., Kuzmina T.A., Bredtmann C.M., Diekmann I., de Carvalho L.M.M., von Samson-Himmelstjerna G., Krücken J. (2021). Genetic variability, cryptic species and phylogenetic relationship of six cyathostomin species based on mitochondrial and nuclear sequences. Sci. Rep..

[79] Ramünke S., de Almeida B.F., von Son-de F.E., von Samson-Himmelstjerna G., Krücken J. (2018). Molecular marker sequences of cattle Cooperia species identify *Cooperia spatulata* as a morphotype of *Cooperia punctata*. PLoS ONE.

[80] Roeber F., Kahn L. (2014). The specific diagnosis of gastrointestinal nematode infections in livestock: Larval culture technique, its limitations and alternative DNA-based approaches. Vet. Parasitol..

[81] Rinaldi L., Krücken J., Martinez-Valladares M., Pepe P., Maurelli M.P., de Queiroz C., Castilla Gómez A.V., Wang T., Cringoli G., Charlier J. (2022). Advances in diagnosis of gastrointestinal nematodes in livestock and companion animals. Adv. Parasitol..

